# Efficacy, safety, and cost-minimization analysis of axicabtagene ciloleucel and tisagenlecleucel CAR T-Cell therapies for treatment of relapsed or refractory follicular lymphoma

**DOI:** 10.1007/s10637-023-01389-w

**Published:** 2023-08-12

**Authors:** Buthainah Ghanem

**Affiliations:** Department of Pharmaceutical Economics and Policy, School of Pharmacy, Chapman University, Irvine, CA USA

**Keywords:** CAR T cell therapy, Non-hodgkin lymphoma, Cancer immunotherapy, Blood cancer, Hematology

## Abstract

Axicabtagene ciloleucel (axi-cel) and tisagenlecleucel (tisa-cel) are chimeric antigen receptor (CAR) T-cell therapies used to treat adult patients with relapsed or refractory follicular lymphoma (rrFL) after two or more lines of systemic therapy. In the absence of head-to-head clinical trials, this study aimed to compare the efficacy, safety, and cost of axi-cel and tisa-cel in the treatment of rrFL after at least two lines of treatment. Overall response rate (ORR) and safety signals were compared using reporting odds ratios (RORs) with 95% confidence intervals (CIs) at p < 0.05. Progression-free survival (PFS), duration of response (DoR), and overall survival (OS) were compared using the Kaplan?Meier method with a log-rank test. Cost and cost-minimization analyses of drug acquisition, drug administration, serious adverse events (AEs), and relapsed management were calculated. Costs were extracted from the IBM-Micromedex Red Book, Centers for Medicare and Medicaid Services, and existing literature. Statistical analyses were conducted using Microsoft Excel and R version 4.0.5. No statistically significant differences were observed between axi-cel and tisa-cel in terms of ORR, DoR, and OS (p > 0.05). PFS was significantly better with tisa-cel (p < 0.05). Axi-cel was significantly associated with higher incidences of CRS, neurologic events, and grade 3–4 AEs than tisa-cel (ROR > 1, p < 0.05). Axi-cel and tisa-cel cost $512,021 and $450,885 per patient, respectively, resulting in savings of US$61,136 with tisa-cel over axi-cel. Tisa-cel appears to have a better safety profile, fewer serious AEs, lower mortality rate, and lower cost than axi-cel.

**Supplementary Information:**

The online version contains supplementary material available at 10.1007/s10637-023-01389-w.

## Key points

Axicabtagene ciloleucel (axi-cel) and tisagenlecleucel (tisa-cel) are recently approved gene therapies for treatment of adult patients with relapsed or refractory follicular lymphoma after two or more lines of treatment. To date, no data were available to understand the differences in their efficacy, safety, and financial impact.

This study compared the two treatments based on pivotal clinical trials.

The results indicated that tisa-cel is a superior treatment over axi-cel, with better efficacy, fewer serious adverse events, and less economic burden.

**Supplementary Information:**

The online version contains supplementary material available at 10.1007/s10637-023-01389-w.

## Introduction

Follicular lymphoma (FL) is the second most common lymphoma diagnosed in the United States (US) and Western Europe, accounting for approximately 35% of all non-Hodgkin lymphomas (NHLs) [1–3]. FL is the most common form of indolent lymphoma, with an estimated incidence rate of 6 new cases per 100,000 persons per year in the US [2, 4]. FL is most frequently diagnosed among people aged 55–64 years and rarely occurs in patients aged under 18 years [4, 5].

Patients with FL respond well to the available chemotherapeutic regimens, with survival rates of 15–18 years [6]. Nevertheless, FL is considered incurable, with approximately 20% of patients experiencing relapse or becoming refractory within 2 years of first-line therapy [7, 8]. Relapsed or refractory FL (rrFL) can be managed with rituximab or second-generation anti-CD20 antibodies, either as a single agent or in combination with chemotherapy, anti-CD20 maintenance therapy, and stem cell transplant [9]. However, successive treatment courses have shown decreased efficacy and durations of remission in most patients with FL [10, 11].

Axicabtagene ciloleucel (axi-cel) and tisagenlecleucel (tisa-cel) are autologous anti-CD19 chimeric antigen receptor (CAR) T-cell therapies, which were recently approved in the US for treatment of adult patients with rrFL after two or more lines of systemic therapy [12, 13]. Pivotal clinical trials—ZUMA-5 for axi-cel and ELARA for tisa-cel—showed high rates of durable responses with relatively manageable safety profiles in extensively pretreated rrFL, including in high-risk patients [14, 15]. ZUMA-5 (axi-cel) was a single-arm, multicenter, phase 2 trial with a median follow-up period of 17.5 months (interquartile range [IQR] 14.1–22.6). Overall response rate (ORR) was observed in 94% of the patients, with 79% having a complete response (CR). The most common grade 3 or worse adverse events (AEs) were neutropenia (61%), anemia (23%), and thrombocytopenia (23%). Grade 3 or higher cytokine release syndrome (CRS) occurred in 7% of the patients, and grade 3 or higher neurological events occurred in 15% of the patients [14]. ELARA (tisa-cel) was a multinational, phase 2 trial with a median follow-up period of 16.6 months (IQR 13.8–20.2). ORR was observed in 89% of the patients, and 74% had CR. Neutropenia (32%), anemia (13%), and decreased white blood cell count (12%) were the most common grade 3 or higher AEs. Grade 3 or higher CRS was not reported, and grade 3 or higher neurological events occurred in 3% of the patients [15]. CAR T-cell therapies are costly. Moreover, the long complicated administrative procedure, serious AEs, and relapse management lead to cost increases. Therefore, mean total healthcare expenditures between CAR T-cell therapies [16, 17].

Likewise, the efficacy, safety, and cost of CAR T-cell therapies vary. Currently, no head-to-head clinical trials have compared these therapies. Thus, this study aimed to compare axi-cel and tisa-cel in terms of efficacy, safety, and treatment cost for adult patients with rrFL after two or more lines of systemic therapy during the first 18–21 months of adoption.

## Methods

The analysis method was based on the pivotal clinical trials of axi-cel (NCT03105336) and tisa-cel (NCT03568461) for adults (≥ 18 years old) with FL, including grade 1–3a FL relapsed or refractory disease after two or more previous lines of therapy. Efficacy, safety, cost, and patient characteristics were compared between these interventions during the first 2 years of treatment.

### Efficacy

Primary and secondary efficacy endpoints were assessed in this analysis. Primary endpoints included ORR, which involved either CR or partial response (PR), stable disease (SD), and progressive disease (PD). Secondary endpoints included progression-free survival (PFS), duration of response (DoR), and overall survival (OS). Efficacy outcomes had the same definition in both clinical trials. ORR was defined as the proportion of patients with the best overall disease response to CR or PR; no response indicated SD or PD in the case of consistent cancer progression [18]. PFS was the time from infusion to disease progression or death from any cause. DoR was the time from first objective response to disease progression or death from any cause. OS was the time from infusion to death from any cause.

In both ZUMA-5 and ELARA, evaluation of primary endpoints was based on reporting odds ratio (ROR) with a 95% confidence interval (CI) at p < 0.05. Kaplan-Meier curves were used to extract secondary endpoints—PFS, DoR, and OS—of the two treatments. Comparative efficacy was assessed using a log-rank test over 18–21 months. Statistical analyses were conducted using Microsoft Excel and R version 4.0.5.

### Safety

RORs with 95% CIs were calculated to assess the disproportionality of grade 3–4 AEs, cytokine release syndrome (CRS), and neurologic events at p < 0.05. Grade 3–4 AEs included neutropenia, anemia, thrombocytopenia, pyrexia, infections, febrile neutropenia, and decreased white blood cell and lymphocyte counts. Statistical analyses were conducted using R version 4.0.5.

### Cost

Treatment cost for each drug was calculated based on pivotal clinical trials. Cost was calculated from the perspective of US healthcare payers and included only direct healthcare costs. Total cost per patient included the cost of drug acquisition, administration, AE management, and retreatment with CAR T-cell therapy (relapsed patients). Wholesale acquisition costs (WACs) of one injection with axi-cel and tisa-cel were extracted from the IBM-Micromedex Red Book [19]. Administration costs of axi-cel and tisa-cel included apheresis, bridging therapy, conditioning chemotherapy (fludarabine 25–30 mg / m^2^ / day, and cyclophosphamide 250–500 mg / m^2^ / day), two IV injections (for conditioning chemotherapy and CAR T-cell therapy), and hospitalization, including in the intensive care unit (ICU). The Centers for Medicare and Medicaid Services (CMS) and previous literature were used to estimate administration costs associated with CAR T-cell therapy [11, 20–23].

Costs of grade 3–4 AEs, which appeared in at least 5% of the patients, and costs of CRS and neurologic events were extracted from the literature and inflated to 2022 USD using the medical care component of the Consumer Price Index from the US Bureau of Labor Statistics [23–27]. Grade 3–4 AEs included neutropenia, anemia, thrombocytopenia, hypoxia, pyrexia, decreased white blood cell count, infections, and febrile neutropenia. AE costs were calculated by multiplying their annual cost by the incidence rate. Eleven patients (8.9%) required a second dose of axi-cel therapy; they were assumed to follow the same treatment regimen as the first dose of axi-cel. No relapse was reported with tisa-cel.

Cost-minimization analyses were performed by calculating incremental costs as percentages between the two drugs. Microsoft Excel was used for all analyses.

### Patient characteristics

Patient characteristics were compared to determine whether these clinical trials were comparable. The patient characteristics were as follows: age (< 65 vs. ≥ 65 years), gender (male vs. female), Eastern Cooperative Oncology Group (ECOG) performance status (0 vs. ≥ 1), disease stage (I/II vs. III/IV), follicular lymphoma international prognostic index (0–2 vs. ≥ 3), high tumor bulk, and previous lines of therapy (phosphoinositide 3-kinase inhibitors [PI3K inhibitor], anti-CD20 monoclonal antibodies [mAb], alkylating agent, lenalidomide, and stem-cell transplantation). The chi-square test (p < 0.05) was used to examine differences between these categorical variables. Statistical analyses were conducted using R version 4.0.5.

## Results

### Efficacy

For the primary endpoints, ORR was slightly higher with axi-cel (94%) than with tisa-cel (89%; Table 1), as CR was slightly higher with axi-cel (79%) than with tisa-cel (74%). PR was the same in both treatments (15%). Nevertheless, RORs showed no significant differences (p > 0.05%) for ORR, CR, and PR. SD appeared to be the same in axi-cel and tisa-cel clinical trials with only 3%, indicating that ROR was insignificant (p = 1). In addition, 8% had PD with the use of the tisa-cel regimen; however, axi-cel showed no PD. Approximately 2% of patients in the axi-cel clinical trial had unknown primary efficacy endpoints, and none of the participants had unknown outcomes in the tisa-cel clinical trial. ROR of PD was significant (p = 0). However, as one clinical trial had zero, the ROR result was indeterminate and could not be interpreted.

**Table 1:** Comparison of efficacy endpoints

Variable	% of patients in ZUMA-5 (axi-cel)	% of patients in ELARA (tisa-cel)	ROR (95% CI)	p-value*
**Primary efficacy endpoints**				
• Overall response rate (ORR)	94%	89%	1.94 (0.69–5.46)	0.20
• Complete response (CR)	79%	74%	1.32 (0.69–2.55)	0.40
• Partial response (PR)	15%	15%	1.00 (0.46–2.17)	1.00
• Stable disease (SD)	3%	3%	1.00 (0.20–5.08)	1.00
• Progressive disease (PD)	0%	8%	0.00 (0.00-NaN)	0.00
• Unknown	2%	0%	Inf (NaN-Inf)	0.16
**Secondary efficacy endpoints**				
	**Number of patients in ZUMA-5 (axi-cel)**	**Number of patients in ELARA (tisa-cel)**	**Time (months)**	p-value**
• Progression-free survival (PFS)	86	94	18	0.02
• Duration of response (DoR)	86	81	21	0.09
• Overall survival (OS)	86	94	18	0.18

* p-value was calculated using the RORs, where axi-cel is the reference

** p-value was calculated using the Kaplan–Meier method with log-rank test over the indicated period

Axi-cel: axicabtagene ciloleucel; Tisa-cel, tisagenlecleucel; ROR, reporting odds ratio; CI, confidence interval

For the secondary efficacy endpoints, the log-rank test showed that PFS was insignificant between the two treatments for month 1–11 (Table 1 and S1). From the 12th month, PFS became significant with better outcomes for tisa-cel. The difference continued to be significant until the end of the data available (18 months). The log-rank test for DoR appeared to be insignificant at the end of the data available (21 months). The difference in DoR was insignificant for month 1–15; however, a significant difference was observed at months 16 and 17 (Table S2). Subsequently, the difference became insignificant until the end of the 21st month. The difference in OS was insignificant between axi-cel and tisa-cel throughout (month by month) and at the end of the 18-month period (Table S3).

### Safety

**Fig. 1:**
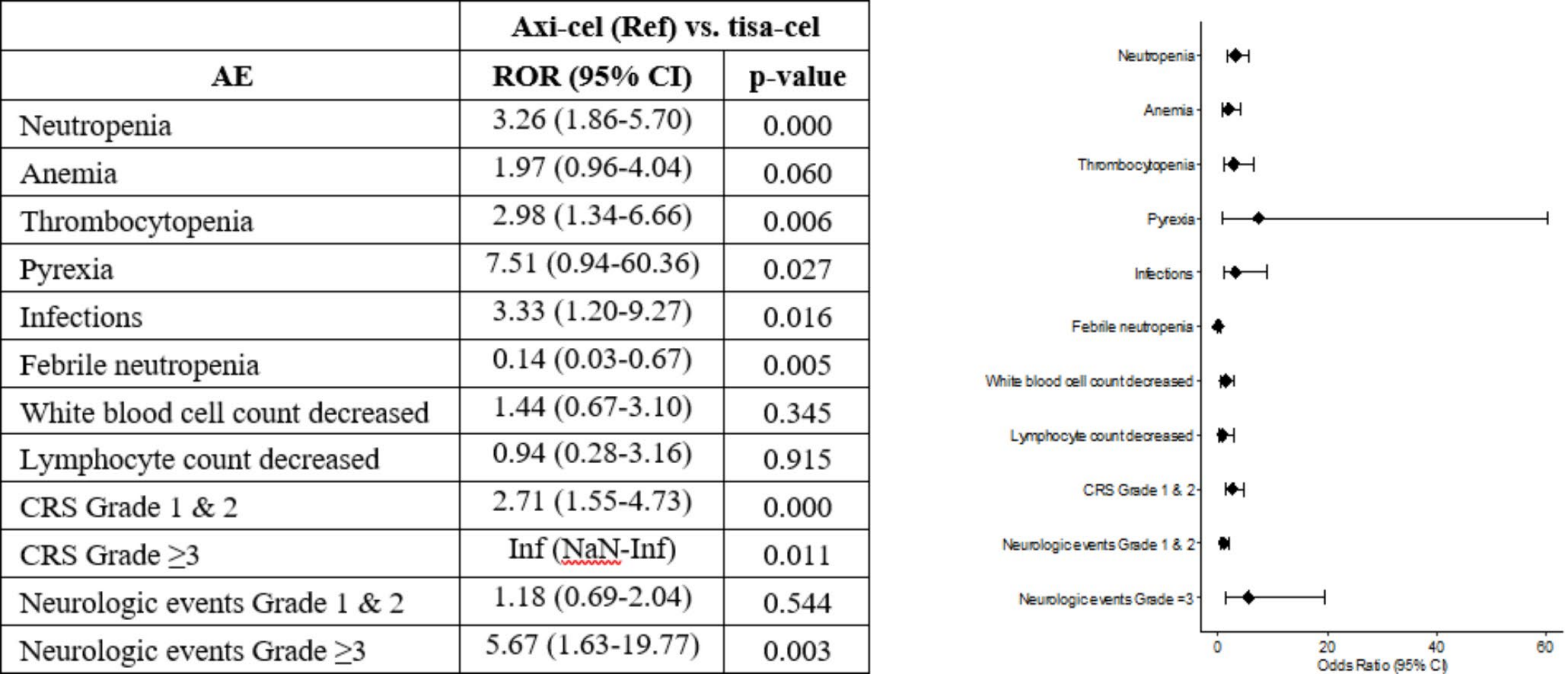
RORs for the CRS, neurologic events, and grade 3–4 AEs associated with CAR T cell therapies used for the treatment of relapsed or refractory follicular lymphoma. CAR T: chimeric antigen receptor-engineered T; Axi-cel: axicabtagene ciloleucel; tisa-cel, tisagenlecleucel; Ref, reference; AE, adverse event; ROR: Reporting Odds Ratio; CI, confidence interval; CRS, cytokine release syndrome

Disproportionality analyses for anemia, decreased white blood cell lymphocyte counts, and grade 1 and 2 neurologic events showed insignificant results between axi-cel and tisa-cel (Fig. 1). Axi-cel was significantly associated with a lower incidence of febrile neutropenia than tisa-cel (RORs < 1, p < 0.05). Conversely, axi-cel was significantly associated with a higher incidence of neutropenia, thrombocytopenia, pyrexia, infections, grade 1 and 2 CRS, and grade 3 or higher neurologic events than tisa-cel (RORs > 1, p < 0.05). Grade 3 or higher CRS was not associated with tisa-cel. Therefore, ROR was infinite.

### Cost

Acquisition costs for one injection of axi-cel and tisa-cel were US$424,000 and US$399,110, respectively (Table 2) [19]. Each patient in the ELARA (tisa-cel) clinical trial needed one injection only. Conversely, in the ZUMA-5 (axi-cel) clinical trial, 8.9% of the patients required a second dose.

**Table 2:** Cost component of CAR T cell therapies used for the treatment of relapsed or refractory follicular lymphoma in the US

		Axi-cel (ZUMA-5)		Tisa-cel (ELARA)		
Variable	Unit cost*	Incidence rate	Cost per patient	Incidence rate	Cost per patient	Reference
**Drug acquisition**			$424,000		$399,110	(IBM Micromedex, 2022)
**Administration**						
Apheresis			$110.39		$110.39	(Centers for Medicare & Medicaid Services, 2022b) (HCPCS code 36511)
Bridging therapy	$3,638.74	3.2%	$117.38	45.4%	$1,650.56	(Simons et al., 2021)
Conditioning chemotherapy						
• Fludarabine (25–30 mg /m^2^/ day)	$92.21	3	$276.64	3	$276.64	(Centers for Medicare & Medicaid Services, 2022a) (HCPCS J9185)
• Cyclophosphamide (250–500 mg /m^2^ /day)	$21.92	15	$328.85	9	$197.31	(Centers for Medicare & Medicaid Services, 2022a) (HCPCS J9070)
IV injection for conditioning chemotherapy			$140.16		$140.16	(Centers for Medicare & Medicaid Services, 2022b) (HCPCS code 96413)
IV infusion for CAR T cell therapy			$140.16		$140.16	(Centers for Medicare & Medicaid Services, 2022b) (HCPCS code 96413)
Hospitalization	$2,868.29	100% for a median of 7 days	$20,087.45	93.8%	$37,982**	(Potnis et al., 2022, Fowler et al., 2022)
Total administration cost			$21,192		$40,497	
**AEs**						
Neutropenia	$7,648	60.5%	$4,626	32.0%	$2,444	(Guzauskas et al., 2018)
Anemia	$7,648	23.4%	$1,789	13.4%	$1,025	(Guzauskas et al., 2018)
Thrombocytopenia	$10,506	23.4%	$2,457	9.3%	$975	(Guzauskas et al., 2018)
Hypoxia	$8,173	6.5%	$527			(National Cancer Institute (NIH), 2022)
Pyrexia	$8,496	7.3%	$617			(Eichten et al., 2021)
White blood cell count decreased	$4,410	16.9%	$747	12.4%	$546	(Potnis et al., 2022)
Infections	$7,642	15.3%	$1,171	5.2%	$394	(Eichten et al., 2021)
Febrile neutropenia	$12,237			10.3%	$1,262	(Eichten et al., 2021)
CRS Grade 1 & 2	$9,289; $5,846	71.8%	$6,667	48.5%	$2,833	(Badaracco et al., 2022)
CRS Grade ≥ 3	$12,210; $21,507	6.5%	$788	0%	$0	(Badaracco et al., 2022)
Neurologic events Grade 1 & 2	$5,168; $3,711	41.1%	$2,125	37.1%	$1,377	(Badaracco et al., 2022)
Neurologic events Grade ≥ 3	$23,461; $13,671	15.3%	$3,595	3.1%	$423	(Badaracco et al., 2022)
Total AEs management cost			$25,109		$11,278	
**Retreatment (relapsed patients)**		8.9%	$41,720	0	$0	
**Total cost per patient (without relapse)**			$470,301		$450,885	
**Total cost per patient (with relapse)**			$512,021		$450,885	

*All costs are inflated to 2022

**Represents the average cost of inpatient and outpatient combined

Axi-cel: axicabtagene ciloleucel; Tisa-cel, tisagenlecleucel; HCPCS, Healthcare Common Procedure Coding System; IV, intravenous; CAR T, chimeric antigen receptor-engineered T; AEs, adverse events; CRS, cytokine release syndrome

The administration procedure started with the apheresis process to harvest peripheral blood mononuclear cells at a target dose of 2 × 10^6^ CAR positive T-cells per kg for axi-cel and (0.6-6) × 10^8^ CAR positive-T cells for tisa-cel. The cost of apheresis was approximately $110.39 according to the Physician Fee Schedule Final Rule [20]. Patients were allowed to receive bridging therapy consistent with their symptoms. A total of 3.2% and 45.4% of the patients received bridging therapy for axi-cel and tisa-cel, respectively. Costs of bridging therapy were estimated based on previous findings of US$3,638.74 for patients undergoing CAR T-cell therapy infusion in the US in 2022 [21]. Bridging therapy was US$117.38 for axi-cel and US$1,650.56 for tisa-cel. Conditioning chemotherapy was conducted one week before infusion of CAR T-cell therapy. Each patient received an intravenous infusion of fludarabine (25–30 mg per m^2^ of body-surface area per day) and cyclophosphamide (250–500 mg per m^2^ per day) on the 5th, 4th, and 3rd days prior to CAR T-cell therapy infusion [22]. The total cost was US$605.49 for axi-cel and US$473.95 for tisa-cel. Hospitalization was the most expensive administrative procedure, costing US$2,868.29 per day [23]. All patients in the ZUMA-5 clinical trial (axi-cel) required hospitalization for a median of 7 days, with a total cost of US$20,087.45 per patient. Approximately 82% of the patients received tisa-cel injections while they were in the hospital, and the rest were outpatients. However, 65% of outpatients required hospitalization after CAR T-cell infusion. The total hospitalization cost was US$37,982 for tisa-cel [11]. Two IV injections were required to administer the conditioning chemotherapy and CAR T-cell therapy, each costing US$140.16 [20]. The total administration cost was US$21,192 for axi-cel and US$40,497 for tisa-cel.

Except for CRS and neurologic events, only grade 3–4 AEs, which occurred in at least 5% of the patients, were included in the analysis. CRS, neurological events, and serious AEs, except for febrile neutropenia, were more intense with axi-cel than with tisa-cel. The total cost of AEs was calculated by multiplying the AE incidence rate by the unit cost for one patient. The total AE cost per patient was US$25,109 for axi-cel and US$11,278 for tisa-cel [23–26]. The mortality rates reported in pivotal clinical trials were 12.1% for axi-cel and 7.2% for tisa-cel.

The total treatment cost was US$470,301 per patient for axi-cel. However, as there was approximately 8.9% relapse with axi-cel, this increased the treatment cost up to US$512,021. Tisa-cel was less costly, with a total cost of US$450,885 per patient. As no relapse was reported with tisa-cel, the treatment cost was lower than that of axi-cel.

The cost-minimization analysis demonstrated that drug acquisition and AE management of axi-cel were associated with US$24,890 and US$13,831 increase in cost, respectively (Table 3), indicating cost increases of 6% and 55%, respectively, with axi-cel over tisa-cel. Administration cost was US$19,305 (91%) lower with axi-cel. Incremental total cost per patient indicated that US$19,416 (4%) would be saved with tisa-cel if there was no relapse, and US$61,136 (12%) with relapse.

**Table 3:** Cost-minimization analyses of CAR T cell therapies used for the treatment of relapsed or refractory follicular lymphoma in the US

Variable	Axi-cel	Tisa-cel	Incremental cost* (%)§
	(ZUMA-5)	(ELARA)	
**Drug acquisition**	$424,000	$399,110	$24,890 (6%)
**Administration cost**	$21,192	$40,497	-$19,305 (-91%)
**AEs management cost**	$25,109	$11,278	$13,831 (55%)
**Total cost per patient (without relapse)**	$470,301	$450,885	$19,416 (4%)
**Total cost per patient (with relapse)**	$512,021	$450,885	$61,136 (12%)

*Incremental cost = cost of axi-cel – cost of tisa-cel

§% = (cost of axi-cel – cost of tisa-cel) × 100%/ cost of axi-cel

CAR T: chimeric antigen receptor-engineered T; Axi-cel: axicabtagene ciloleucel; Tisa-cel, tisagenlecleucel; AEs, adverse events

### Patient characteristics

The chi-square test showed no statistically significant differences by age (< 65 vs. ≥ 65 years), gender (male vs. female), ECOG performance status (0 vs. ≥ 1), disease stage (I/II vs. III/IV), high tumor bulk, and previous lines of therapy (Table 4). Only the follicular lymphoma international prognostic index showed a statistically significant difference between axi-cel and tisa-cel (p < 0.05). This difference was in favor of axi-cel; 44% of the patients in the axi-cel group had a higher risk than that in the tisa-cel group (59.8%).

**Table 4:** Comparison of patient characteristics between ZUMA-5 and ELARA clinical trials for relapsed or refractory follicular lymphoma

Variable	Axi-cel ZUMA-5	Tisa-cel ELARA	p-value*
Age (years)			
• Median (IQR)	60 (53–67)	57 (49–64)	
• ≥ 65, n (%)	38 (31)	24 (24.7)	0.3324
Gender, n (%)			0.28
• Male	73 (59)	64 (66)	
• Female	51 (41)	33 (34)	
ECOG performance status, n (%)			0.4349
• 0	78 (63)	56 (57.7)	
• ≥ 1	46 (37)	41 (43.3)	
Disease stage, n (%)			0.9861
• I or II	18 (15)	14 (14.4)	
• III or IV	106 (85)	83 (85.6)	
Follicular lymphoma international prognostic index, n (%)			0.01652
• 0–2	70 (57)	39 (40.2)	
• ≥ 3	54 (44)	58 (59.8)	
High tumor bulk^δ^, n (%)	64 (52)	62 (63.9)	0.06671
Previous lines of therapy, n (%)			0.1523
• Previous PI3K inhibitor	34 (27)	20 (20.6)	
• Previous anti-CD20 mAb and alkylating agent	123 (99)	97 (100)	
• Previous lenalidomide	38 (31)	21 (21.6)	
• Previous stem-cell transplantation	30 (24)	35 (36.1)	

*X^2^ test at p < 0.05. ^δ^Bulky disease was defined as a nodal or extranodal tumor mass > 7 cm in diameter or the involvement of three or more nodal sites with diameters > 3 cm

Axi-cel: axicabtagene ciloleucel; Tisa-cel, tisagenlecleucel; IQR, interquartile range; n, number; ECOG: Eastern Cooperative Oncology Group; PI3K: Phosphoinositide 3-kinase inhibitors; mAb, monoclonal antibodies

## Discussion

FL is one of the most difficult diseases to treat and is generally considered incurable. Although patients respond well to initial treatment, they tend to relapse or become refractory multiple times during their lifetime [28, 29]. There is no consensus on the best treatment option for patients with rrFL [30]. Advanced treatment strategies, such as PI3K inhibitors, stem cell transplantation, radiotherapy, and chemotherapy followed by rituximab maintenance therapy, have been shown to improve outcomes, such as OS, PFS, and quality of life (QoL), in rrFL patients [31, 32]. Nevertheless, the majority of patients relapse or become refractory and require multiple lines of treatment [33]. Therefore, new therapeutics are required to achieve control with minimal drug-related toxicity. The introduction of axi-cel and tisa-cel CAR T-cell therapy in rrFL patients has shown promising outcomes with manageable safety profiles. In the absence of a head-to-head clinical trial, this analysis compared the efficacy, safety, cost, and patient characteristics of axi-cel and tisa-cel for treatment of adult patients with rrFL after two or more lines of systemic therapy.

The analysis showed no statistically significant differences between the two treatment regimens in terms of ORR, CR, PR, or SD (p > 0.05). The median PFS, DoR, and OS were not reached in either clinical trial. Patients in the ELARA (tisa-cel) trial showed a statistically better PFS than those in the ZUMA-5 (axi-cel) trial starting from the 12th month. DoR was not significant after 21 months of follow-up. However, there was a significant difference between the 16th and 17th months, indicating that short-term follow-up is insufficient. Long term follow-up throughout the treatment course is required to understand and compare efficacy endpoints. OS was insignificant over 18 months follow-up. There is currently no efficacy analysis comparing axi-cel and tisa-cel in rrFL; however, Bachy et al. found that axi-cel was significantly associated with better ORR, PFS, and OS than tisa-cel for relapsed or refractory diffuse large B-cell lymphoma after two or more previous lines of treatment [34]. The same study demonstrated no statistically significant differences in terms of DoR. This difference could be justified, as the two diseases are subtypes of NHL with different characteristics and clinical aggressiveness (i.e., indolent, aggressive, and highly aggressive) [35, 36]. The two diseases are usually managed using different protocols, suggesting that a direct comparison between them might not be accurate even if the same drugs are used [37].

Except for febrile neutropenia, axi-cel was significantly associated with a higher incidence of serious AEs. These findings are consistent with previous studies [34, 38, 39]. Therefore, axi-cel might not be the best option for severely ill or elderly patients.

This analysis demonstrated that using tisa-cel over axi-cel for rrFL could provide a comparable efficacy profile at lower costs. Previous cost-effectiveness analysis models found that axi-cel was superior to tisa-cel for relapsed or refractory diffuse large B-cell lymphoma at a lower or minimally higher cost [17, 40]. Although this study did not use a cost-effectiveness analysis model, it measured the short-term value-cost relationship. Comparison of different diseases could be one of the reasons for the difference in outcomes. Moreover, the cost-effectiveness analysis model was conducted when the costs of axi-cel and tisa-cel were the same at US$373,000, whereas axi-cel costs increased, with incremental cost of US$24,890 over tisa-cel, in the present study [17].

Patient characteristics were compared between ZUMA-5 and ELARA clinical trials to identify statistically significant differences that could affect study outcomes. The results revealed that only the follicular lymphoma international prognostic index was statistically significant. Approximately 59.8% of the patients had ≥ 3 (high risk) in the ELARA trial compared to 44% in the ZUMA-5 trial. The follicular lymphoma international prognostic index was used to predict FL survival rates in patients receiving chemotherapy [41–43]. Consequently, patients in the ELARA study had lower odds of better outcomes than those in the ZUMA-5 study. In addition, all characteristics were comparable between the two trials.

This analysis indicated that using tisa-cel over axi-cel for rrFL after two or more lines of systemic therapy could provide a comparable efficacy profile for ORR, DoR, and OS, a better efficacy profile for PFS, lower mortality rate, lower incidence of serious AEs, and US$61,136 savings over the first 18 months after adoption. However, further long-term research with a larger sample size is required to confirm these findings.

## Limitations

This study was based on data derived from two separate clinical trials with different populations and inclusion and exclusion criteria. The main limitation of this analysis was the absence of a head-to-head clinical trial. WAC was used to represent acquisition costs of axi-cel and tisa-cel, which did not include rebates, co-payments, or discounts. Moreover, this analysis only estimated direct healthcare costs and did not include indirect costs.

## Conclusions

The use of axi-cel and tisa-cel CAR T-cell therapies for rrFL after two or more lines of treatment appeared to be effective with manageable safety profiles. Axi-cel was associated with a comparable efficacy profile, more serious AEs, and higher cost than tisa-cel. However, further research with head-to-head clinical trials and patient follow-up over the treatment course is required to understand and compare efficacy and safety profiles, as well as to assess the economic impact of these CAR T-cell therapies.

### Electronic supplementary material

Below is the link to the electronic supplementary material.

Supplementary Material 1
