# Supplementary material for: Efficacy, safety, and cost-minimization analysis of axicabtagene ciloleucel and tisagenlecleucel CAR T-Cell therapies for treatment of relapsed or refractory follicular lymphoma
Source: Invest New Drugs. 2023 Aug 12;41(5):710–8. doi: 10.1007/s10637-023-01389-w (PMC10560186; doi:10.1007/s10637-023-01389-w)
Supplement: Supplementary file 1 — Supplementary Material 1 [file 10637_2023_1389_MOESM1_ESM.docx]

**Appendix**

Table S1. The Log Rank Test comparing progression-free survival Kaplan-Meier curves.

| **Time (months)** | **Axi-cel** | | | **Tisa-cel** | | | **Total patients at risk** | **Total number of events** | **Expected number of events in axi-cel** | **Expected number of events in tisa-cel** | **p-value** |
| --- | --- | --- | --- | --- | --- | --- | --- | --- | --- | --- | --- |
|  | **PFS (%)** | **PFS (n)** | **Events (n)** | **PFS (%)** | **PFS (n)** | **Events (n)** |  |  |  |  |  |
| 0 | 100 | 86 | 0 | 100 | 94 | 0 | 180 | 0 | 0.17 | 0.19 | 0.53 |
| 1 | 100 | 86 | 0 | 100 | 94 | 0 | 179 | 0 | 0.22 | 0.25 | 0.94 |
| 2 | 98 | 85 | 1 | 100 | 93 | 0 | 178 | 2 | 0.74 | 0.81 | 0.69 |
| 3 | 90 | 77 | 7 | 100 | 83 | 10 | 161 | 17 | 8.24 | 8.86 | 0.75 |
| 4 | 88 | 76 | 1 | 99 | 82 | 1 | 158 | 3 | 1.37 | 1.48 | 0.79 |
| 5 | 88 | 76 | 0 | 98 | 79 | 2 | 155 | 2 | 1.15 | 1.20 | 0.47 |
| 6 | 81 | 70 | 6 | 91 | 68 | 11 | 138 | 17 | 8.81 | 8.61 | 0.17 |
| 7 | 81 | 69 | 0 | 88 | 68 | 0 | 137 | 1 | 0.42 | 0.41 | 0.17 |
| 8 | 81 | 69 | 0 | 89 | 68 | 0 | 137 | 0 | 0.00 | 0.00 | 0.17 |
| 9 | 80 | 69 | 0 | 87 | 67 | 0 | 136 | 1 | 0.42 | 0.41 | 0.17 |
| 10 | 79 | 68 | 1 | 87 | 67 | 0 | 135 | 1 | 0.54 | 0.54 | 0.23 |
| 11 | 79 | 68 | 0 | 85 | 65 | 2 | 133 | 2 | 0.96 | 0.93 | 0.14 |
| 12 | 77 | 66 | 2 | 83 | 50 | 16 | 116 | 17 | 9.88 | 7.44 | 0.00 |
| 13 | 72 | 62 | 4 | 81 | 50 | 0 | 112 | 4 | 2.21 | 1.77 | 0.01 |
| 14 | 70 | 60 | 2 | 79 | 49 | 0 | 110 | 2 | 1.33 | 1.09 | 0.01 |
| 15 | 70 | 60 | 0 | 77 | 49 | 0 | 109 | 0 | 0.26 | 0.21 | 0.01 |
| 16 | 68 | 58 | 2 | 75 | 48 | 0 | 107 | 2 | 1.20 | 0.99 | 0.01 |
| 17 | 68 | 58 | 0 | 73 | 49 | 0 | 107 | 0 | 0.00 | 0.00 | 0.02 |
| 18 | 68 | 58 | 0 | 73 | 49 | 0 | 107 | 0 | 0.00 | 0.00 | 0.02 |

Axi-cel: axicabtagene ciloleucel; Tisa-cel, tisagenlecleucel; PFS, progression-free survival; n, number.

Table S2. The Log Rank Test comparing duration of response Kaplan-Meier curves.

| **Time (months)** | **Axi-cel** | | | **Tisa-cel** | | | **Total patients at risk** | **Total number of events** | **Expected number of events in axi-cel** | **Expected number of events in tisa-cel** | **p-value** |
| --- | --- | --- | --- | --- | --- | --- | --- | --- | --- | --- | --- |
|  | **DoR (%)** | **DoR (n)** | **Events (n)** | **DoR (%)** | **DoR (n)** | **Events** |  |  |  |  |  |
| 0 | 100 | 86 | 0 | 100 | 81 | 0 | 167 | 0 | 0.00 | 0.00 |  |
| 1 | 99 | 85 | 1 | 99 | 80 | 1 | 165 | 2 | 0.98 | 0.92 | 0.87 |
| 2 | 93 | 80 | 5 | 96 | 78 | 2 | 158 | 7 | 3.63 | 3.52 | 0.42 |
| 3 | 89 | 77 | 4 | 92 | 75 | 3 | 151 | 7 | 3.50 | 3.40 | 0.49 |
| 4 | 89 | 76 | 0 | 85 | 69 | 6 | 145 | 6 | 3.14 | 2.84 | 0.55 |
| 5 | 86 | 74 | 3 | 83 | 67 | 2 | 141 | 5 | 2.39 | 2.17 | 0.63 |
| 6 | 85 | 73 | 1 | 82 | 66 | 0 | 139 | 1 | 0.59 | 0.54 | 0.67 |
| 7 | 85 | 73 | 0 | 81 | 66 | 1 | 139 | 0 | 0.23 | 0.21 | 0.52 |
| 8 | 84 | 73 | 1 | 80 | 64 | 1 | 137 | 2 | 1.03 | 0.91 | 0.47 |
| 9 | 84 | 73 | 0 | 77 | 62 | 2 | 135 | 2 | 1.13 | 0.97 | 0.27 |
| 10 | 81 | 70 | 3 | 72 | 58 | 4 | 128 | 6 | 3.54 | 2.95 | 0.18 |
| 11 | 80 | 69 | 1 | 72 | 58 | 0 | 127 | 1 | 0.63 | 0.54 | 0.26 |
| 12 | 75 | 65 | 4 | 72 | 58 | 0 | 123 | 4 | 2.13 | 1.92 | 0.59 |
| 13 | 74 | 64 | 1 | 71 | 58 | 1 | 121 | 2 | 0.94 | 0.85 | 0.63 |
| 14 | 73 | 63 | 1 | 70 | 57 | 1 | 120 | 2 | 0.87 | 0.78 | 0.61 |
| 15 | 72 | 62 | 1 | 68 | 55 | 1 | 118 | 2 | 1.18 | 1.04 | 0.53 |
| 16 | 72 | 62 | 0 | 55 | 45 | 10 | 107 | 11 | 6.31 | 4.56 | 0.04 |
| 17 | 72 | 62 | 0 | 55 | 45 | 0 | 106 | 0 | 0.11 | 0.08 | 0.03 |
| 18 | 70 | 60 | 2 | 55 | 44 | 0 | 104 | 2 | 1.16 | 0.86 | 0.05 |
| 19 | 70 | 60 | 0 | 54 | 44 | 0 | 104 | 0 | 0.23 | 0.17 | 0.05 |
| 20 | 70 | 60 | 0 | 54 | 44 | 0 | 104 | 0 | 0.00 | 0.00 | 0.05 |
| 21 | 67 | 58 | 3 | 54 | 44 | 0 | 101 | 3 | 1.55 | 1.18 | 0.09 |

Axi-cel: axicabtagene ciloleucel; Tisa-cel: tisagenlecleucel; DoR: duration of response; n: number.

Table S3. The Log Rank Test comparing overall survival Kaplan-Meier curves.

| **Time (months)** | **Axi-cel** | | | **Tisa-cel** | | | **Total patients at risk** | **Total number of events** | **Expected number of Events in axi-cel** | **Expected number of events in tisa-cel** | **p-value** |
| --- | --- | --- | --- | --- | --- | --- | --- | --- | --- | --- | --- |
|  | **OS (%)** | **OS (n)** | **Events (n)** | **OS (%)** | **OS (n)** | **Events (n)** |  |  |  |  |  |
| 0 | 99 | 85 | 1 | 100 | 94 | 0 | 180 | 0 | 0.02 | 0.02 |  |
| 1 | 99 | 85 | 1 | 100 | 94 | 0 | 179 | 1 | 0.24 | 0.27 |  |
| 2 | 98 | 84 | 1 | 100 | 94 | 0 | 179 | 1 | 0.24 | 0.27 | 0.05 |
| 3 | 98 | 84 | 0 | 100 | 94 | 0 | 179 | 0 | 0.00 | 0.00 | 0.05 |
| 4 | 98 | 84 | 0 | 100 | 94 | 0 | 179 | 0 | 0.00 | 0.00 | 0.05 |
| 5 | 97 | 84 | 1 | 100 | 94 | 0 | 178 | 1 | 0.63 | 0.70 | 0.10 |
| 6 | 96 | 83 | 1 | 99 | 93 | 1 | 176 | 2 | 0.94 | 1.06 | 0.19 |
| 7 | 96 | 82 | 0 | 97 | 91 | 2 | 174 | 2 | 0.94 | 1.04 | 0.67 |
| 8 | 96 | 82 | 0 | 96 | 90 | 1 | 173 | 1 | 0.46 | 0.50 | 0.95 |
| 9 | 96 | 82 | 0 | 95 | 89 | 1 | 171 | 1 | 0.67 | 0.72 | 0.69 |
| 10 | 94 | 81 | 2 | 95 | 89 | 0 | 170 | 1 | 0.51 | 0.56 | 0.78 |
| 11 | 94 | 81 | 0 | 95 | 89 | 0 | 170 | 0 | 0.05 | 0.05 | 0.76 |
| 12 | 94 | 81 | 0 | 95 | 89 | 0 | 170 | 0 | 0.16 | 0.18 | 0.89 |
| 13 | 93 | 80 | 1 | 95 | 89 | 0 | 169 | 1 | 0.48 | 0.53 | 0.65 |
| 14 | 91 | 78 | 2 | 95 | 89 | 0 | 167 | 2 | 0.95 | 1.08 | 0.31 |
| 15 | 89 | 77 | 1 | 95 | 89 | 0 | 166 | 1 | 0.47 | 0.54 | 0.21 |
| 16 | 89 | 77 | 0 | 94 | 88 | 1 | 165 | 1 | 0.44 | 0.50 | 0.32 |
| 17 | 89 | 77 | 0 | 94 | 88 | 0 | 165 | 0 | 0.00 | 0.00 | 0.27 |
| 18 | 88 | 76 | 1 | 94 | 88 | 0 | 164 | 1 | 0.47 | 0.54 | 0.18 |

Axi-cel: axicabtagene ciloleucel; Tisa-cel, tisagenlecleucel; OS, overall survival; n, number.
